# Health-related quality of life among children/adolescents living with HIV/AIDS in Lagos State, Nigeria

**DOI:** 10.4314/ahs.v23i2.13

**Published:** 2023-06

**Authors:** Kazeem Osuolale, Abideen Salako, Adesola Musa, Oluwatosin Odubela, Fatimah Adepoju-Olajuwon, Agatha David, Tiitilola Gbaja-Biamila, Oliver Ezechi, Babatunde Salako

**Affiliations:** 1 Biostatistics Subunit, Grant, Monitoring and Evaluation Unit, Department of Statistics. University of Ibadan Nigeria; 2 Clinical Sciences Department, Nigerian Institute of Medical Research

**Keywords:** Quality of life, Kruskal-Wallis analysis, children

## Abstract

Health-Related Quality of Life (HRQL) is commonly used to assess the impact of health status on quality of life. The HRQL data obtained does not follow the assumption of normality and a non-parametric test was used to make inference in this study. This study compares the characteristics of children with HRQL classified as good, intermediate, and poor quality of life. The children and adolescents that have a good health-related quality of life had a mean rank of 75.5, intermediate, 27.0 while children and adolescents with poor HRQL had a mean rank of 8.5. The health -related quality of life differs significantly across the demographic characteristics. However, tertiary education does not differ significantly on HRQL. The Kruskal-Wallis's chi-squared was 76.95 with two degrees of freedom and p-value < 2.2e-16. The p-value < 0.05 indicates sufficient evidence that HRQL of children and adolescents differs significantly across the three categories. Conclusively, children and adolescents in the three categories have different quality of life. It is clear that most of the children and adolescents had a good health-related quality of life once they have been taking their drugs regularly as prescribed by the physician.

## Introduction

Human immunodeficiency virus/Acquired Immunodeficiency Syndrome (HIV/AIDS) continues to be one of the serious health challenges affecting the world. The burden of the disease still remains a major public health problem in low- and middle-income countries including Nigeria 1 despite the provision of antiretroviral therapy (ART) during pregnancy, breastfeeding, encouraging mitigations, increased awareness, and the remarkable improvement in treatment coverage as noted by Salako, Agatha and Babasola, et al. 2. There were about 38million people living with HIV worldwide as at 2020, out of which 1.7 million were children less than 15 years old. It was reported in 2020 that more than 600,000 people died of HIV/AIDS related illnesses in the world 3. However, the population of children with HIV surviving into adolescence and later adulthood is on the rise in sub-Sa-haran Africa due to the availability of comprehensive universal care which includes ART 4-7.

People living with HIV (PLHIV) in Nigeria were more than two million as at 2019 with approximately 45,000 AIDS-related death in the country 8. Over 200,000 of the HIV cases were adolescents (between the ages of 10-19), accounting for 7% of the total number of people with HIV in Nigeria 9. Lagos state was listed as one of the six states that made up the 41% of the PLHIVs in Nigeria 10. Although, the new HIV infections among children (<15 years) was reported to have reduced more than 50% globally, much more are still required to be done to improve knowledge of HIV and HIV testing among adolescents and young people 9. HIV-related stigma and discrimination persists in all the areas of life despite knowledge and awareness. Stigma affects the social, educational, and behavioural lifestyle of everyone especially PLHIV 11. Regardless of the most vulnerable populations (children), the impact of HIV/AIDS at the family level incredibly trickles down to them 12. Paediatric treatments of HIV largely depend on the CD4 count in developing countries, hence infected children are solely assessed and treated based on the CD4 lymphocyte count. However, due to the massive psychosocial challenges encountered by children living with HIV (CLHIV), the entire disease impact is not usually portrayed using such clinical markers, this leads to a significantly lower health-related quality of life (HRQL) for people living with HIV than the general population, despite that the majority of the PLHIV are immunologically stable 13.

Health-Related Quality of Life (HRQL) is an appraisal of the state of an individual's health status in relation to the modern theory of healthcare. It reveals the physical, psychological, social and emotional well-beings of the patients 14. The HRQL considers many other aspects such as the psychological, emotional and social impact of the disease and not only the disease. The HRQL have been used by clinicians and public health specialists to provide wide-ranging evaluation of the burden of preventable diseases, injuries, treatments and short- and long-term disabilities 15 - 16. The population's health could be assessed in terms of improving the quality of lives through the measure of health outcomes and not just by saving lives alone 17. The process of measuring quality of life provides opportunity to assess intervention from the patients' point of view 18. Enhancing the quality-of-life of HIV-infected children is the spotlight of recent management of children living with HIV (CLHIV). Therefore, the overall effect of HIV infection on the lives of CLHIV can be systematically assessed using the health-related quality of life (HRQL) measure as a substitute to the conventional clinical assessment of health. This will also inform policy-makers better concerning the different needs of the vulnerable populations. Lack of information on the HRQL of children and adolescent living with HIV is a key issue in Nigeria. In addition, several methods have been used to assess the quality of life of HIV/AIDS patients by several researchers in the previous studies with their limitations. The impact of HIV/AIDS on adolescents' quality of life has been reported by those authors. Some authors reported on the negative impact of the disease on life, other researchers showed positive impact of HIV/AIDS on the lives of young people. Kruskal-Wallis's test was used in this study to evaluate the health-related quality of life among children/adolescents living with HIV /AIDS since the HRQL data in this study do not conform with normality assumptions. This test is one distribution-free test which is analogous to the one-factor ANOVA F-test developed by Kruskal and Wallis 19 and is used to compare or estimate the statistical differences of multiple independent groups on a continuous or ordinal dependent variable. This study, therefore assesses the health-related quality of life among children/adolescents living with HIV /AIDS in Lagos State, Nigeria.

## Methodology

### Study Design and Period

This is a retrospective study to evaluate the health-related quality of life among children/adolescents living with HIV /AIDS. This study was a cross sectional design that utilized data obtained from a study carried out between May 2019 and July 2019 at the Paediatric and Adolescent Clinic, Clinical Sciences Department at the Nigerian Institute of Medical Research Yaba, Lagos State, Nigeria.

### Study Population

The HRQL of 113 children and adolescent aged 8-18 years living with HIV/AIDS who have been on ART for at least three months was used in this study. Data of children with co-morbid illness (Cerebral Palsy, Seizure disorders, Sickle cell anaemia or other chronic co-morbid illness such as Hepatitis B/C), all newly diagnosed ALHIV less than 3months on ART therapy and those with acute illness were not included in this study.

### Study Procedure

Children and adolescents attending the clinic were randomly selected from the clinic database and assessed for eligibility for the study. The study was described to those who meet the study criteria and their caregivers by the clinician and designated counsellor among the study team members. Those in agreement to take part in the study were taken through a detailed informed consent and assent process and thereafter they were enrolled into the study. The case record form (CRF) was used to collect data on sociodemographic characteristic (age, sex, level of education and parental HIV status among others. The age at diagnosis, current/previous antiretroviral therapy, length of time on ART, and current CD4 count and viral load were obtained from the clinic database. The quality-of-life assessment for the study participants was done using a pre-tested interviewer-administered questionnaire and the Paediatric quality of life questionnaire (PedsQL™) 20 already validated amongst children of various cultures and countries including those from Africa, Nigeria inclusive 21-23. Prior to the commencement of the main study, permission to use the questionnaire was obtained from copyright owners 20.

The scale was scored and linearly transformed to a 0-100 scale as follows: 0=100, 1=75, 2=50, 3=25, 4=0.The absolute Health related quality of life was represented as mean. The Overall/Physical/Psychosocial HRQL aggregate score was categorized into good (81 - 100), intermediate (60 - 80) and poor (31.0 – 59.9) quality of life.

## Statistical Methods and Data Analysis

Participants demographic characteristics were summarised using frequency count and percentage with their respective p-values. The Kruskal- Wallis test was used to assess the statistical differences in the distribution between the groups. The test statistics (K-W) used is given as 19:


(1) $$H=\{12/[N(N+1)]\}\sum_{i=1}^{k}\left(R_{j}^{2}/n_{j}\right)-3(N+1)$$

Where,

**n_j_ =** number of data values in **j^th^** column

**N** = total number of data values, equal to the sum of the n_j_ over j

**K** = number of columns (levels)

**Rj** = sum of ranks of the data in **j^th^** column

However, there is one additional step as a correction to H for the number of ties. This

corrected *H, Hc*, is now given as:


(2) $$H_{c}=H/\left\lfloor 1-\sum \left(r^{3}-r\right)/\left(N^{3}-N\right)\right\rfloor$$

where *r* = the number of tied observations. Assuming the null hypothesis is true, the test statistic H (or Hc) has a distribution that is well approximated by a chi-square (X^2^ ) distribution with degree of freedom K-1. The analysis in this study was not manually computed but done using R software with Kruskal. Test package. A p-value of less than 0.05 was considered statistically significant in this study. The choice of this test was based on the fact that the HRQL data obtained in this study do not conform with normality assumptions.

## Results and Discussion of Findings

This section presents the results analysed using non-parametric statistical method. The results were presented in Table 1 through Table 3. Table 1 shows the demographic characteristics of the participants while results on the health-related quality of life of the children and adolescents based on their demographic characteristics is given in Table 2. A total of 108 children and adolescents participated in the study. A greater percentage of 69 (63.9%) of the respondents were between 13 and 18 years of age. Fifty-seven (52.8%) of the participants were males while 51 (47.2%) were females. Most 81 (75.0%) of the respondents had a secondary school while only 4 (3.7%) had a tertiary level of education. More than half of the participants 94 (87.0%) were Christians while 14 (13.0%) were Muslims.

**Table 1:** Demographic Characteristics of the Participants

Variable	Frequency (n)	Percentage (%)
**Age group**		
**8 - 12**	39	36.1
**13 - 18**	69	63.9
**Gender**		
Female	51	47.2
Male	57	52.8
**Education**		
Primary	23	21.3
Secondary	81	75.0
Tertiary	4	3.7
**Religion**		
Christian	94	87.0
Muslim	14	13.0

**Table 2:** Health-related Quality of Life according to Demographic Characteristics of the Participants

Variable	Health-related Quality of life			K-W H	p value
Age group	Good n (%)	Intermediate n C%	Poor n (%)		
**8 - 12**	22 (28.9)	9 (47.4)	8 (50.0)	30.42	**0.0001**
**13 - 18**	54 (71.1)	10 (52.6)	8 (50.0)	40.83	**0.0001**
**Gender**					
Female	26 (34.2)	14 (73.7)	12 (75.0)	43.06	**0.0001**
Male	50 (65.8)	5 (26.3)	4 (25.0)	22.69	**0.0001**
**Education**					
Primary	12 (15.8)	6 (31.6)	5 (31.3)	18.31	**0.0001**
Secondary	61 (80.3)	13 (68.4)	10 (62.5)	50.84	**0.0001**
Tertiary	3 (3.9)	-	1 (6.3)	1.80	0.180
**Religion**					
Christian	66 (86.8)	16 (84.2)	15 (93.8)	65.04	**0.0001**
Muslim	10 (13.2)	3 (15.8)	1 (6.3)	8.19	0.017

**Table 3:** Description of the Health-related quality of life of Children and Adolescents based on the ART line

**ART level**	**Health-related Quality of life**				**K-W H (p-value)**
	**Good (%)**	**Intermediate (%)**	**Poor (%)**	**Total (%)**	**76.95 (<2.2e^-16^)**
**Line 1**	53 (65.4)	14 (17.3)	14 (17.3)	**81 (71.7)**	
**Line 2**	23 (71.9)	7 (21.9)	2 (6.2)	**32 (28.3)**	
**Total**	**76**	**21**	**16**	**113**	
**Mean Rank**	75.5	27.0	8.5		

The health -related quality of life differs significantly across the age group, gender, educational status and religion (p<0.05). However, tertiary education does not differ significantly on the health- related quality of life of children and adolescents (p>0.05). This may possibly be due to the fact that only few of the participants used in the study has attained tertiary level of education.

The results presented in Table 3 shows the health-related quality of life of the children and adolescents based on the line of ART they are. It can be seen that 81 (71.7%) of the children are on the ART line 1 out of which 53 (69.7%) had good HRQL and 17.3% had intermediate and poor HRQL. More than one-quarter (28.3%) were on the line 2 of ART out of which 23 (71.9%) had good HRQL, 21.9% had intermediate and 6.2% had poor HRQL. The children and adolescents that have a good health-related quality of life had a mean rank of 75.5, intermediate health-related quality of life had the mean rank of 27.0 while children and adolescents with poor health-related quality of life had a mean rank of 8.5. Among the 113 children and adolescents used in this study, 81 (71.7%) of them were on the first-line drugs out of which 53 (65.4%) of the children had good health-related quality of life while 14 (17.3%) were recorded separately for intermediate and poor health-related quality of life. Besides, 32 (28.3%) of 113 children and adolescents used in this study were on the second line drugs out of which 23 (71.9%) of the children had a good health-related quality of life while 7 (21.9%) of the children and adolescents had intermediate and only 2 (6.2%) had a poor health-related quality of life.

The Kruskal-Wallis statistic in Table 3 was 76.95 with two degrees of freedom (df) and p-value < 2.2e-16 (Table 3). The p-value obtained is less than 0.05 indicating that there is sufficient evidence that the health-related quality of life of children and adolescents differs significantly across the three categories. The overall quality of life of children living with HIV in this study was good and their quality of life differs based on ART level. This positive result is due to the good immunological status, the completeness of the services of the clinic specialized in paediatric/adolescent HIV; through easy access to ART at no cost, immediate attention to problems, routine counselling services, social support groups through regular teen club meetings, and parental support. This finding is consistent with previous work 24 - 26. Thus, the benefits of improved care characterized by the ease of availability and accessibility of ART are affirmed.

## Conclusion

In our study we found that children and adolescents with a good health-related quality of life had the highest mean rank (75.5). This implies that a larger percentage of the children and adolescents has good health-related quality of life. The study has also established that the health -related quality of life differs significantly across age group, gender, educational status and religion (p<0.05). However, tertiary education does not differ significantly on the health- related quality of life of children and adolescents. The Kruskal-Wallis chi-squared was 76.954 with two degree of freedom (df) and p-value < 2.2e-16. The p-value obtained is less than 0.05 indicating that there is sufficient evidence that the health-related quality of life of children and adolescents differs significantly across the three categories.

It is evident in this study that most of the children and adolescents had a good health-related quality of life once they have been taking their drugs regularly as prescribed by the physician. The analysis and findings in this study are based on the categorization of the health-related quality of life into good, intermediate and poor quality of life.

The limitation of the current study includes the cross-sectional nature of the main study and our inability to identify causality for participants with good, intermediate or poor health-related quality of life. Therefore, we will adopt the longitudinal study/focus group discussion in future studies and identify possible predisposing factors or causes, specific psychosocial problems in children living with HIV with the appropriate tool.
